# The complete mitochondrial genome of *Nadezhdiella cantori* (Hope, 1843) (Coleoptera: Cerambycidae)

**DOI:** 10.1080/23802359.2022.2131368

**Published:** 2022-10-27

**Authors:** Hongmin Tian, Zhu Li

**Affiliations:** College of Plant Protection, Southwest University, Chongqing, China

**Keywords:** Cerambycinae, *Cerambycini*, longhorn beetle, mitogenome, phylogenetic analysis

## Abstract

The complete mitochondrial genome of *Nadezhdiella cantori* (Hope, 1843) is 16,049 bp in length, containing 13 protein-coding genes (PCGs), 22 transfer RNA genes (tRNAs), two ribosomal RNA genes (rRNAs), and an A-T rich region (control region). The gene order is conserved and identical to most other previously sequenced Cerambycidae. Phylogenetic analysis showed that the newly sequenced *N. cantori* was in a well-supported clade tribe Cerambycini, subfamily Cerambycinae. These results support the currently accepted taxonomy and provide a better understanding of the phylogenetic analysis of the Cerambycidae.

## Introduction

Family Cerambycidae, known as longhorn beetles or longicorn beetles, contains over 30,000 species worldwide. There are more than 12,000 species described in subfamily Cerambycinae, the second largest subfamily. The phylogenetic relationship of Cerambycinae remains unclear (Lee and Lee 2020). A recent study suggested that mitochondrial genomics are useful for revealing phylogenetic relationships (Nie et al. 2021). According to the data from GenBank, the complete mitogenomes of 27 species of Cerambycinae have been reported so far. *Nadezhdiella cantori* (Hope, 1843), belonging to subfamily Cerambycinae, is an important citrus pest that feeds on the trunks and main branches of citrus trees (Wang and Zeng 2004) and is widely distributed in China, Laos, Thailand, and Vietnam. However, the complete mitochondrial genome of *N. cantori* has not been reported. In the present study, the characterization of the mitogenome of *N. cantori* is reported.

## Materials and methods

**Figure 1.**
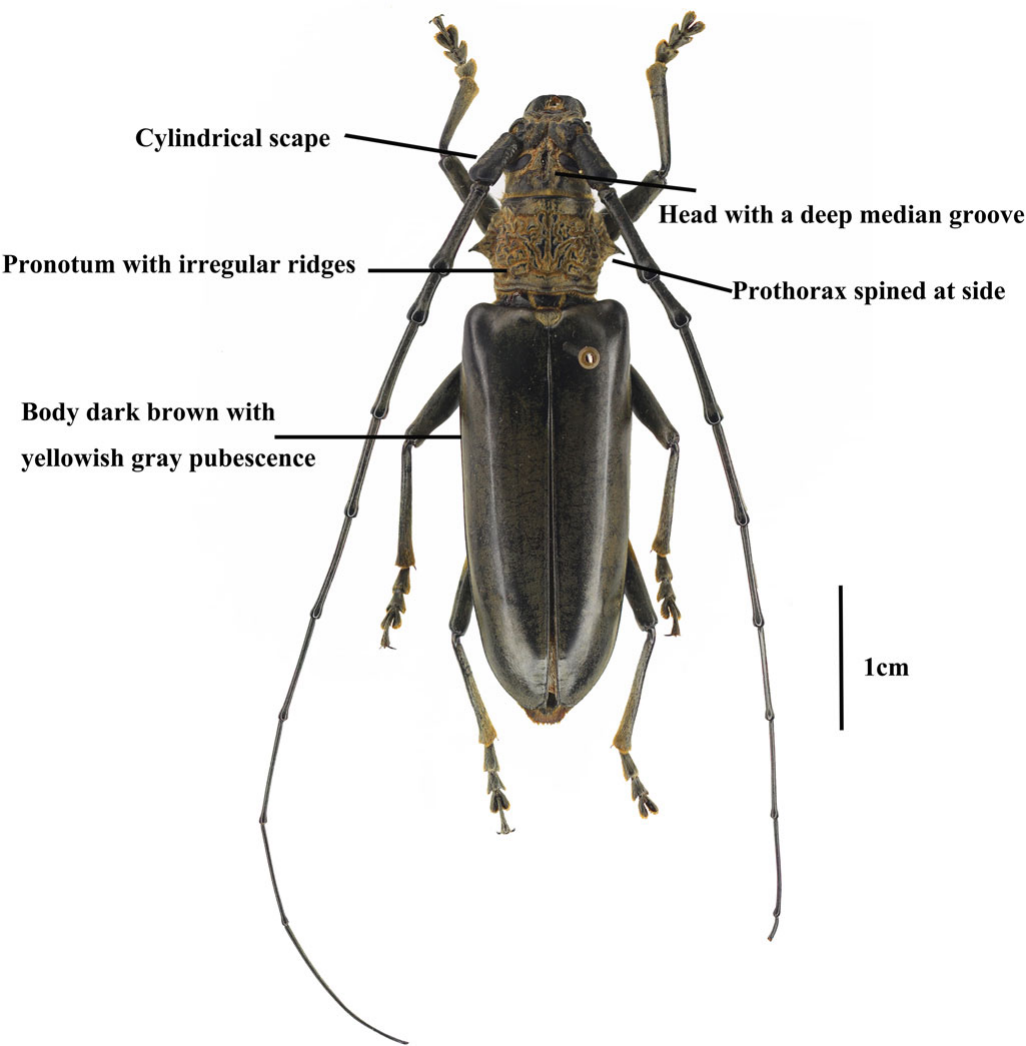
Adult habitus of *Nadezhdiella cantori* (Hope, 1843).

Specimens of *N. cantori* were collected from Jinyun Mountain National Nature Reserve, Chongqing, China (106°22′E, 29°49′N) on 6 June 2021 and were deposited at the Entomological Collection of Southwest University, Chongqing, China (voucher number SWU-01021601. Zhu Li, email: Lizhu0526@swu.edu.cn). The sample was preserved in 100% ethanol at −20 °C (Figure 1).

Genomic DNA was extracted from an adult’s muscle tissue of the prothorax or leg by the CTAB method (Reineke et al. 1998). Genomic DNA was subjected to pin-end (PE) sequencing based on the Illumina NovaSeq sequencing platform using next-generation sequencing technology at Personalbio (Shanghai, China). The data obtained by sequencing were used to assemble the mitochondrial genome using GetOrganelles (Jin et al. 2020). The assembled mitochondrial genome was annotated by using MITOS Web Server (Bernt et al. 2013) and Geneious (Kearse et al. 2012). Bayesian’s inference was performed using MrBayes v.3.2 (Ronquist et al. 2012) with the best model of the gene fragment GTR + F+I + G4 (*atp6, cox1, cox2*, *cox3, cytb, nad1, nad2, nad3*, *nad4, nad4L*, and *nad5*), and HKY + F+I + G4 (*atp8* and *nad6*) was determined by ModelFinder (Kalyaanamoorthy et al. 2017).

## Results

**Figure 2.**
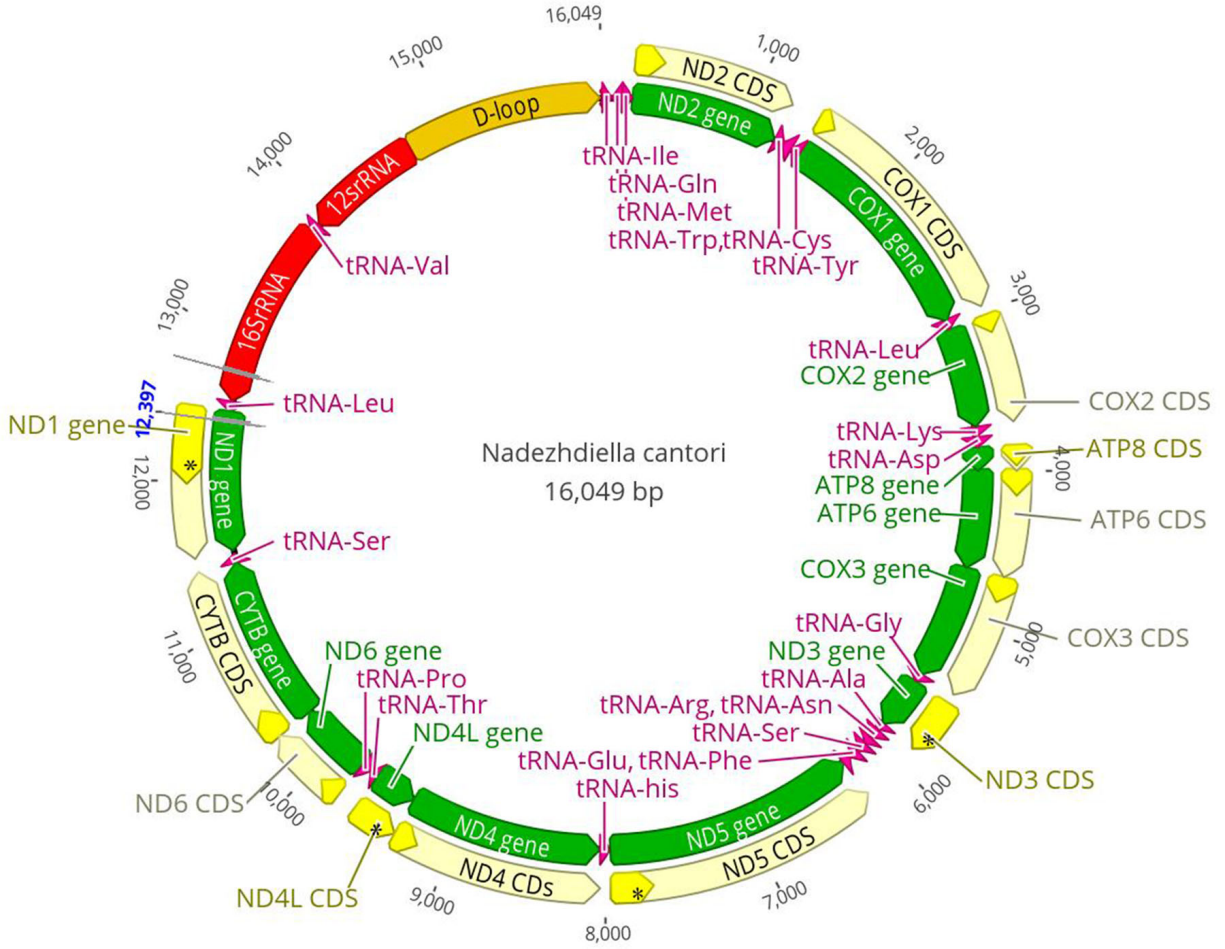
Genome map of the mitogenome of *N. cantori.*

The complete mitogenome of *N. cantori* (GenBank accession number NC_061180) is 16,049 bp in length, containing the entire set of 37 genes usually present in most insect mtDNAs (13 protein-coding genes (PCGs), 22 transfer RNA genes, and two ribosomal RNAs), and a putative control region (Figure 2). Fourteen genes were transcribed on the minority strand (N-strand), whereas the others were oriented on the majority strand (J-strand). The overall base composition of the *N. cantori* mitogenome was A (40.1%), T (31.9%), C (17.2%), and G (10.8%), with a high A + T bias of 72.00%.

A total of 31 bp intergenic spacer were dispersed in six locations, ranging from 1 to 22 bp in length. The longest interval between *trnS2* and *nad1* was 22 bp. Gene overlaps were found at 14 gene junctions and involved a total of 47 bp, the longest 8 bp overlap located between *trnY* and *cox1*. The length of 22 tRNAs varied between 64 bp (*trnT*, *trnH*, *trnF*, *trnE*, *trnR*, *and trnG*) and 71 bp (*trnK*), comprising a total of 1456 bp. The length of *rrnL* is 1289 bp with an A + T content of 76%, and *rrnS* is 804 bp with an A + T content of 70.5%. The 1356 bp control region is located between *rrnS* and *trnI* and has a remarkably high A + T content (81.8%).

Twelve PCGs begin with ATN start codons, excluding *nad1*, which begins with TTG: ATG for *cox3*, *nad4*, *nad4l*, *atp6*, and *cytb*; ATT for *cox1*, *nad2*, and *nad6*; ATA for *nad3* and *nad5*; ATC for *cox2* and *atp8*. The PCGs terminate with a TAN (TAA or TAG) stop codon, whereas five PCGs (*cox2*, *cox3*, *nad3*, *nad4*, and *nad5*) terminate with an incomplete stop codon (T–). For the seven other PCGs, TAAs (*nad2*, *nad6*, *atp8*, *atp6*, *nad4L*, and *cox1*) or TAGs (*cytb and nad1*) were used.

**Figure 3.**
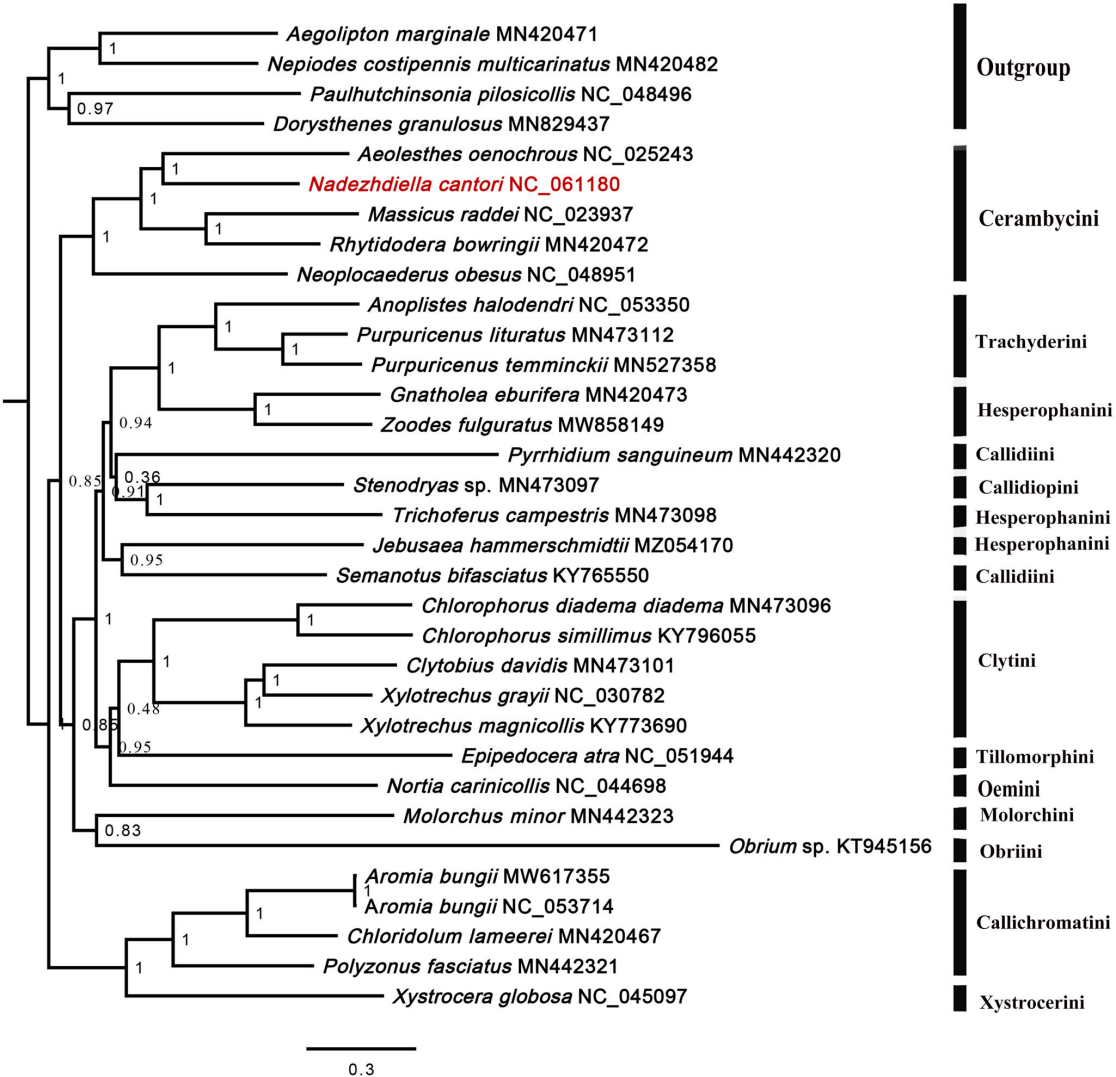
Bayesian tree of 28 species of Cerambycinae, including *Nadezhdiella cantori* (in this study, NC_061180) and 4 outgroups based on the sequence of 13 mitochondrial protein-coding genes. Numbers on each node are posterior probabilities.

We reconstructed the phylogenetic relationships of 28 Cerambycinae species based on 13 PCGs. The Bayesian tree (Figure 3) showed that Cerambycinae is monophyletic. Most tribes are monophyletic, whereas Hesperophanini and Callidiini are not. *N. cantori* formed a sister to *Aeolesthes oenochrous* with high value support.

## Discussion and conclusions

This study determined the gene sequence of *N. cantori* and analyzed the main characteristics of its mitochondrial genome. The mitochondrial genome is conserved in terms of gene composition, location, and direction, which are the same as other previously sequenced mitochondrial genomes of Cerambycidae (Yang et al. 2019; Li et al. 2021).

Phylogenetic analysis showed that Cerambycinae is monophyletic. Hesperophanini and Callidiini are not, which is consistent with the previous studies (Lee and Lee 2020; Nie et al. 2021). In its present phylogenetic position, *N. cantori* is clustered within *Cerambycini* species, consistent with its classification based on morphological data. The mitogenome of *N. cantori* will help with molecular identification and population genetic studies of the species. It also provides a basis for establishing the phylogenetic relationships of Cerambycinae.

## Data Availability

The mitogenome sequence data that support the findings of this study are openly available in GenBank of NCBI at https://www.ncbi.nlm.nih.gov/ under the accession no. NC_061180. The associated BioProject, SRA, and Bio-Sample numbers are PRJNA835288, SRR19143081, and SAMN28100428, respectively.
